# Cross-Sectional Observational Study of Typical in utero Fetal Movements Using Machine Learning

**DOI:** 10.1159/000528757

**Published:** 2022-12-20

**Authors:** Lana Vasung, Junshen Xu, Esra Abaci-Turk, Cindy Zhou, Elizabeth Holland, William H. Barth, Carol Barnewolt, Susan Connolly, Judy Estroff, Polina Golland, Henry A. Feldman, Elfar Adalsteinsson, P. Ellen Grant

**Affiliations:** ^a^Department of Pediatrics, Boston Children's Hospital, and Harvard Medical School, Boston, Massachusetts, USA; ^b^Department of Electrical Engineering and Computer Science, MIT, Cambridge, Massachusetts, USA; ^c^Department of Obstetrics and Gynecology, Massachusetts General Hospital, and Harvard Medical School, Boston, Massachusetts, USA; ^d^Department of Radiology, Boston Children's Hospital, and Harvard Medical School, Boston, Massachusetts, USA; ^e^Computer Science and Artificial Intelligence Laboratory, MIT, Cambridge, Massachusetts, USA; ^f^Institute for Medical Engineering and Science, MIT, Cambridge, Massachusetts, USA; ^g^Institutional Centers for Clinical and Translational Research, Boston Children's Hospital, Boston, Massachusetts, USA

**Keywords:** Fetal movement, In utero fetal magnetic resonance imaging, Maternal hyperoxia

## Abstract

Early variations of fetal movements are the hallmark of a healthy developing central nervous system. However, there are no automatic methods to quantify the complex 3D motion of the developing fetus in utero. The aim of this prospective study was to use machine learning (ML) on in utero MRI to perform quantitative kinematic analysis of fetal limb movement, assessing the impact of maternal, placental, and fetal factors. In this cross-sectional, observational study, we used 76 sets of fetal (24–40 gestational weeks [GW]) blood oxygenation level-dependent (BOLD) MRI scans of 52 women (18–45 years old) during typical pregnancies. Pregnant women were scanned for 5–10 min while breathing room air (21% O<sub>2</sub>) and for 5–10 min while breathing 100% FiO<sub>2</sub> in supine and/or lateral position. BOLD acquisition time was 20 min in total with effective temporal resolution approximately 3 s. To quantify upper and lower limb kinematics, we used a 3D convolutional neural network previously trained to track fetal key points (wrists, elbows, shoulders, ankles, knees, hips) on similar BOLD time series. Tracking was visually assessed, errors were manually corrected, and the absolute movement time (AMT) for each joint was calculated. To identify variables that had a significant association with AMT, we constructed a mixed-model ANOVA with interaction terms. Fetuses showed significantly longer duration of limb movements during maternal hyperoxia. We also found a significant centrifugal increase of AMT across limbs and significantly longer AMT of upper extremities <31 GW and longer AMT of lower extremities >35 GW. In conclusion, using ML we successfully quantified complex 3D fetal limb motion in utero and across gestation, showing maternal factors (hyperoxia) and fetal factors (gestational age, joint) that impact movement. Quantification of fetal motion on MRI is a potential new biomarker of fetal health and neuromuscular development.

## Introduction

Fetal behavior, defined as any observable action or reaction to external stimuli, reflects the function and maturation of the central nervous system. Simple fetal movements start as early as seven gestational weeks (GW). By 10 GW, complex spontaneous movements, characterized by continuous exchange of numerous combinations of flexions-extensions, abductions-adductions, and rotations, emerge [1]. These movements are called general movements and are believed to be an early hallmark of a healthy developing brain [2] which can assist in the early diagnosis of neurodevelopmental disorders [3]. Finally, assessment of the amount of fetal movements in utero is often used in non-stress test and biophysical profiles to evaluate fetal well-being and guide labor management.

It has been estimated that at least half of 4 million women who give birth in the USA receive supplemental oxygen and are subjected to supraphysiologic oxygen concentrations [4]. The supplemental oxygen is often administered in the absence of evidence of maternal hypoxemia to improve the fetal metabolic milieu [5]. However, clear guidelines regarding the indication, duration, and dosage of supplemental oxygen therapy during pregnancy or labor are virtually non-existing [6]. Moreover, the lack of large-scale randomized controlled trials and longitudinal studies, which could address the magnitude of the effect of oxygen usage on mother and fetus, contrasts a growing number of small cross-sectional and cohort studies addressing the physiologic changes caused by chronic maternal hyperoxygenation in conditions associated with maternal or fetal hypoxia [7, 8, 9]. Thus, the physiological effects of short-term administration of supplemental oxygen to pregnant mothers, in the absence of fetal or maternal hypoxia, remain unknown. In this study, besides characterizing the amount of fetal movement, we also aimed to explore the effects of short-term maternal hyperoxia on the duration of fetal movement.

Magnetic resonance imaging (MRI), due to its large field of view, superior tissue contrast, and ability to capture the movement of all four fetal extremities simultaneously over time, has the potential to be superior to ultrasound for the unbiased and non-subjective analysis of whole-body fetal movements throughout gestation. The purpose of our study was to assess the ability of our recently developed convolutional neural network [10] to detect changes in fetal extremity movement. We hypothesized that maternal, placental, and fetal factors would have a quantitative impact on the duration of fetal limb movement.

## Material and Methods

### Subjects

For this prospective observational cross-sectional study, we screened and recruited 81 pregnant women in the prenatal clinic at Massachusetts General Hospital and Brigham and Women's Hospital in Boston between April 2017 and May 2018. Inclusion criteria were pregnant mothers with no major underlying medical condition between 18 and 45 years of age, singleton pregnancies with no medical complication of the fetus during pregnancy, and fetal age between 24 and 40 GW. Exclusion criteria were chromosomal anomalies, known genetic disorders, or major congenital malformation of the fetus, presence of any condition or abnormality that in the opinion of the investigator would compromise the safety of the mother or fetus, quality or completeness of the data, pregnant mothers with contraindication to MRI (such as a pacemaker, metal in the body, exceeding scanner weight or bore diameter limits [550 lbs, 70 cm]) or claustrophobia. Written informed consent was obtained from all subjects. This study protocol was reviewed and approved by Boston Children's Hospital IRB, approval number 00012586. All methods were carried out in accordance with institutional guidelines and regulations.

### Magnetic Resonance Imaging

All MRI scans were performed on the same 3T Skyra scanner (Siemens Healthineers, Erlangen, Germany). Blood oxygenation level-dependent (BOLD) images of the whole uterus were acquired using multislice, single-shot, gradient-echo EPI sequence with an in-plane resolution of 3 × 3 mm^2^, slice thickness of 3 mm acquired in interleaved order, mean matrix size = 120 × 120 × 80, TR = 5–8 s, TE = 32–38 ms, and FA = 90°. To improve the temporal resolution for kinematic measurements, each image was separated into two sub-volumes (i.e., the effective time interval between EPI volumes was 2.5–4 s). The maternal oxygenation was adjusted to provide an initial 5–10 min of room air (21% O_2_), followed by 5–10 min of 100% FiO_2_ via a non-rebreathing facial mask.

### Kinematic Measurements

To analyze the kinematics of upper and lower limbs, using 76 BOLD MRI datasets we randomly chose 1,705 volumes and manually labeled twelve fetal key points (wrists, elbows, shoulders, ankles, knees, hips), and trained a 3D convolutional neural network (4) to predict the locations of these key points given the input 3D MR volume. We used a 3D UNet-based network to predict the locations of key points in the input MR volume. Exploiting the idea of heatmap prediction in human pose estimation, the network generates a volume representing the per-pixel probability for each key point, and the location with the highest probability is used as the predicted key point location. The target heatmaps are Gaussian distributions centered at the ground truth locations of key points. The network is trained to minimize the mean squared error between the predicted and target heatmaps. Afterward, the trained network is applied to the unlabeled dataset to predict fetal poses. We then visually assessed and manually corrected the predicted results. The correct fetal pose time series were used for further analysis. Only the training data are randomly chosen to make sure it has a distribution similar to the whole dataset. Then, the trained model is used to predict the key points in all the unlabeled frames.

In total, 1.92% of the key points were corrected. The mean difference between the predicted and corrected key points was 2.64 mm, while the median difference was 2.26 mm, which was less than the in-plane resolution. Kinematic measurements were used to compute the absolute movement time (AMT). For each key point, we calculated spatial coordinates (*p*^*x*^_*i*_, *p*^*y*^_*i*_, *p*^*z*^_*i*_)at the *i*th time frame. We then computed the velocity of the key point by using the

**Figure:**
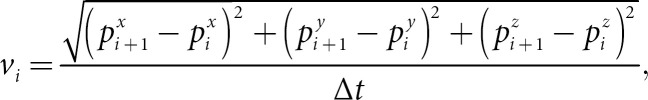

where Δ*t* is the time interval between two frames. To compute the AMT in seconds, we defined key point as moving if the velocity *v*_*i*_is greater than a threshold *v*_*th*_. Finally, the AMT was measured as

**Figure:**
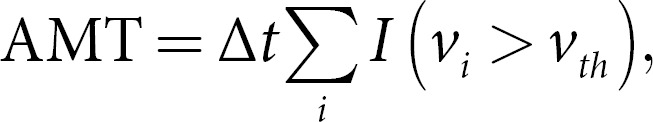

where *I* is the indicator function, i.e., *I*(*v*_*i*_ > *v*_*th*_) is 1 if *v*_*i*_ > *v*_*th*_, otherwise, it is 0. We set *v*_*th*_ = 3 mm/s (1 pixel/s).

### Statistical Analyses

Demographic information (Table 1) and parameter estimates were reported using count, percentage, mean, and standard deviation (SD). The kinematic measure of joint movements (AMT) exhibited skewed distributions and was log-transformed for analysis. To identify variables that have a significant association with the AMT, we constructed a mixed-model ANOVA with interaction terms. The choice of the ANOVA parametric method was confirmed by examining and verifying a quasi-Gaussian distribution of log-transformed AMT residuals. All our mixed-effect linear models were appropriate for the sample size enabling us to correctly estimate the statistical precision and form valid inferences. We employed robust regression to detect and reduce the influence of outliers [11]. Each data point was assigned a weight according to its deviation from the fitted model, using the bisquare function with 99% efficiency, and the model was re-fitted iteratively until the weights stabilized.

Based on the literature, we hypothesized that the dependent variable AMT is explained by maternal factors (the position of the mother during MRI scan [12] [left lateral, right lateral, supine, or tilt]; parity [13] [first pregnancy, multipara, or unknown]); and oxygen environment [14] [normoxia or hyperoxia]), placental factors (placental position [15] [anterior, posterior, superior, or previa]), and fetal factors {fetal sex [16] [male, female]; fetal position [17] [left occiput, left sacrum, occiput anterior, right occiput, or right sacrum]; fetal age group [18] [rounded to the nearest week]; joint factors (distance from the core of the body [proximal, middle, or distal]; extremity [upper or lower]; and side of the body [left or right])}. We included additional terms to allow potential interactions between the extremity and fetal age group, and the side of the body and fetal age group representing our hypotheses. Finally, to account for repeated measurements of a given mother and repeated scans of the mother in a given position, two random effects were added to the model, one specific to the subject and one specific to the repeated scans of a subject in a given position. The fitted region-specific coefficients of log AMT were retransformed to yield estimates of the effects on AMT in percentage (%). To calculate the false discovery rate (FDR), we imposed a maximum type I error rate of 5% on each fixed effect or interaction, applying the 5% error rate to a family of closely related hypotheses.

## Results

### Maternal, Placental, and Fetal Characteristics

**Fig. 1:**
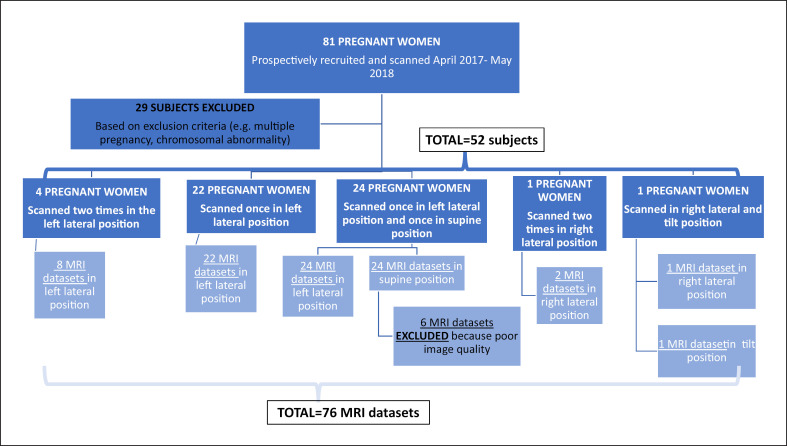
Flowchart of our study sample shows inclusion and exclusion.

From 81 screened pregnant women scanned between April 2017 and May 2018, 29 were excluded (22 due to the multiple pregnancies, 7 due to the overall poor image quality, Fig. 1). Of 52 pregnant women with singleton pregnancies that were finally included in this study, a total of 76 sets of fetal MRI scans were obtained.

Of 52 subjects, 19 subjects were scanned in two maternal positions (i.e., 18 of them in left lateral and supine positions and 1 of them in left lateral and tilted positions). Within 52 subjects, for 5 subjects, two separate BOLD datasets were collected at the same MRI session in the same maternal position (i.e., four were in the left lateral position and one in right lateral position). The rest of the subjects were scanned in the left lateral position and only one BOLD dataset was collected. For each subject, the first BOLD dataset was acquired at least 15 min after maternal positioning. For the subjects with two different positions, after the first BOLD acquisition, the mother was brought out of the scanner by moving the table and her position changed. Mothers did not stand or sit at any point while changing positions. Maternal repositioning and structural data acquisition for anatomical reference in the second position ensured at least 20 min between the BOLD datasets with oxygen exposure after the first position. The maternal, placental, and fetal characteristics of the studied population can be found in Table 1.

### Kinematic Measures: AMT

#### Maternal Factors

**Fig. 2:**
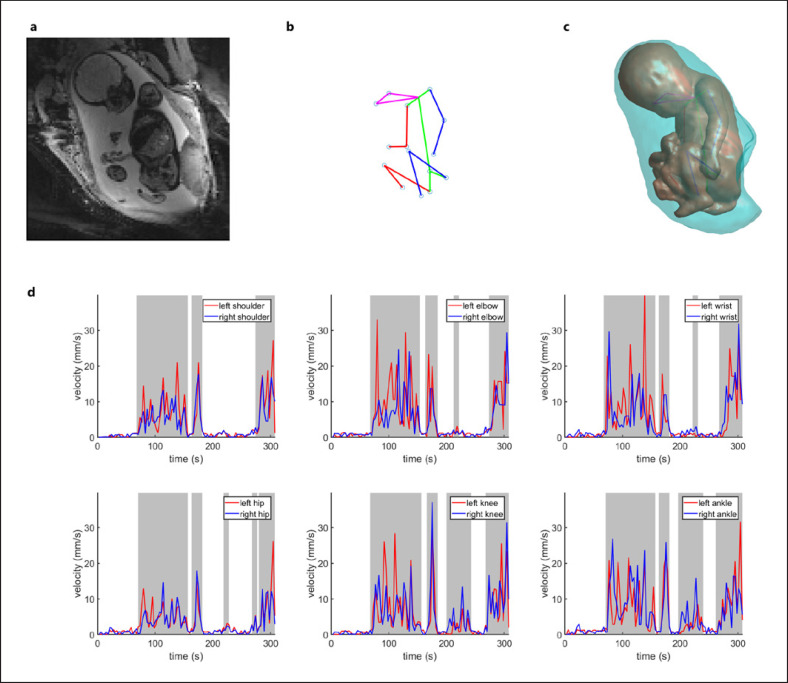
Difference in AMT between lower and upper extremities before 30 GW. **a** An example slice of the MR volume. **b** The fetal pose generated from labeled key points. **c** 3D masks of the fetal body and uterus. **d** The change of velocity over time, shaded regions indicate velocities are greater than the threshold.

**Fig. 3:**
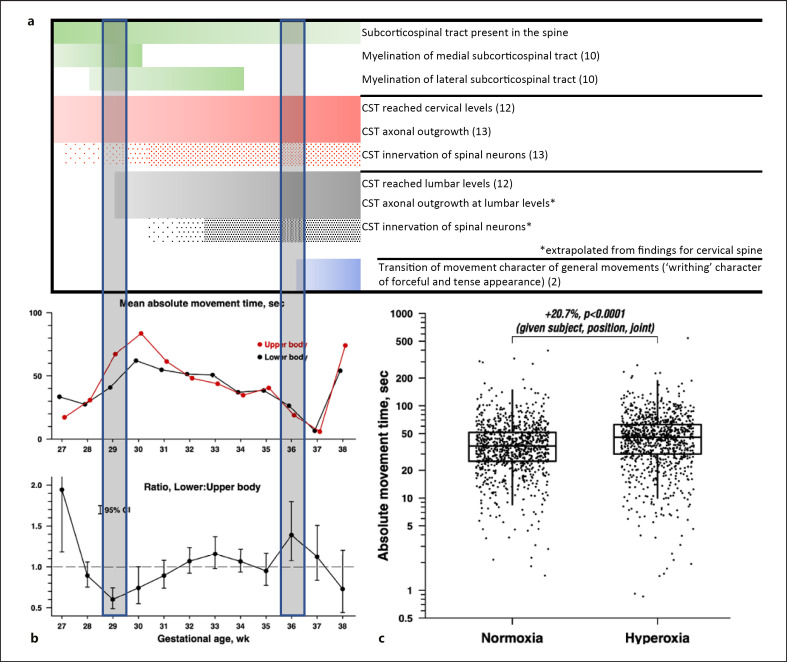
Overview of major histogenic events (**a**), scatterplots showing the mean AMT (upper row) and difference in AMT between upper and lower extremities for gestational age (bottom row) (**b**), and boxplots showing the difference in AMT between hyperoxia and normoxia (**c**). **a** Neurogenic events relevant for movement. **b** Upper row: mean AMT for upper and lower extremities, by fetal age group (rounded to the nearest week), averaged across all subjects, joints, and other covariates. Lower row: ratio between the movements of upper and lower extremities (difference in %) and 95% confidence intervals for the fetal age group, for a given subject, joint, and other covariate values. Shaded vertical bars mark periods during which significant differences between movements of upper and lower extremities were found, as indicated by non-overlap of confidence interval with dashed horizontal line at ratio 1.0. **c** The data points are adjusted for the variables in the model. Middle quartile/median (the horizontal line through the box); quartile boundaries (top and the bottom of the box). “Whiskers” extend to the farthest data point within the 1.5 × interquartile range of the box. For a given subject, joint, and other covariate values, the mean absolute movement time was 20.7% higher in the hyperoxic state (95% CI 13.9–27.9%, *p* < 0.0001).

During maternal hyperoxia, compared to normoxia, fetuses showed a 20.7% longer duration of movements (SE = 3.6, 95% CI = 13.9–27.9, *p* < 0.0001, FDR <0.0001, Table 2; Fig. 2, 3). We did not find an association between parity and the mother's position during the MRI scan and AMT. Moreover, in order to confirm that data from the single tilt position scan and data acquired in the right lateral position did not skew the larger dataset and affect our results we performed a sensitivity analysis, repeating the entire ANOVA while retaining only the data from left lateral and supine positions. The sample size of measurements was reduced minimally, from 1,824 to 1,728, and the findings were virtually unchanged with respect to magnitude and statistical significance, including the contrast between the two positions.

#### Placental Factors

We did not find an association between any placental factors and AMT.

#### Fetal Factors

AMT showed a difference between joints, with distal joints (i.e., wrists and ankles: the joints of the limbs that are the most distal from the core body) exhibiting 20.6% (SE = 4.5, 95% CI = 12.4–29.5, *p* < 0.0001, FDR < 0.0001) longer movement time compared to the middle joints (elbows and knees), and middle joints exhibiting 23.8% (SE = 4.6, 95% CI = 15.3–32.9, *p* < 0.0001, FDR < 0.0001, Table 2) longer movement time compared to the proximal joints (shoulders and hips). We found shorter AMT of lower extremities compared to upper extremities at particular gestational ages (interaction *p* < 0.0001). Figure 3b illustrates the 39.3% shorter movement of lower extremities between 28.5 and 29.5 GW (SE = 6.8, 95% CI = [−50.7]−[−25.3], *p* < 0.0001, FDR < 0.0001), 25.7% shorter movement of lower extremities between 29.5 and 30.5 GW (SE = 12.2, 95% CI = [−44.9]−[−0.2], *p* = 0.05, FDR = 0.154), and 39.4% longer movement of lower extremities between 35.5 and 36.5 GW (SE = 19.4, 95% CI = 7.9–80, *p* = 0.011, FDR = 0.04). Finally, we did not observe any significant differences in AMT between males and females.

## Discussion

To our knowledge, this is the first prospective study using machine learning (ML) on fetal MRI to yield an unbiased assessment of fetal limb movement duration in utero. Our results provide the first evidence that maternal hyperoxia, rather than maternal and placental position during the MRI scan, plays a role in the early fetal limb movement. We also found a centrifugal increase in fetal joint movement and evidence that the pattern of movements is different between upper and lower extremities between <31 GW and >35 GW.

There is a paucity of data describing the effects of maternal hyperoxia in healthy fetuses. During normal pregnancy, a compensatory increase in the duration of fetal movements during hyperoxia might lead to the consumption of a larger amount of oxygen without creating a substantial amount of free radicals [19]. Human studies have shown that maternal hyperoxygenation leads to an increase in fetal oxygenation and after 31 weeks increased blood flow to the fetal lungs [20]. Direct ultrasound observation of fetal movements has failed to detect an increase in fetal motion after maternal hyperoxia during normal pregnancy. However, these studies were limited by a small number of subjects, less sensitive methods for detection of fetal motion, low statistical power, and confounding factors [14, 21, 22] limiting the generalizability of these findings. This is the first human study to confirm increased fetal motion with maternal hyperoxia. However, animal and human studies that could provide enough evidence to elucidate the magnitude and the physiologic effects of short-term maternal hyperoxia on a fetus during typical pregnancy do not exist. Here we provide evidence that short-term maternal hyperoxia during the third trimester of typical pregnancy is associated with a significantly longer duration of limb movements. These results suggest that administration of supplemental oxygen, in the absence of evidence of maternal hypoxemia, might have an effect on the results of non-stress tests or biophysical profiles (directly by increasing the amount of fetal movement), or fetal monitoring during labor (indirectly via changes in heart rate caused by an increase in fetal movement). However, dedicated animal studies, as well as longitudinal studies and large-scale randomized controlled trials in humans, are needed to elucidate the physiological effects of maternal hyperoxia on fetuses.

As expected, our results show a centrifugal increase in the amount of joint movement (displacement) across limbs which could be explained by the additive joint effect resulting from multijoint movement coordination [23]. Significantly longer AMT of the upper compared to the lower limbs between 28.5 and 30.5 GW might reflect the maturation of the fetal nervous system (Fig. 3a, b). Monoaminergic bulbospinal pathways are present in the entire spinal cord at around 6 GW [24]. However, myelination of the bulbospinal “subcorticospinal” pathways occurs between 20 and 34 GW [25], in parallel with histochemical differentiation of the human muscle [26]. In contrast to bulbospinal pathways, the pyramidal tract shows protracted growth. The first corticofugal axons arise from the immature cortical plate during the embryonic period [<8 GW [27]]. The cortico-spinal axons reach the caudal medulla and pyramidal decussation by the end of the embryonic period [28] which is followed by a long waiting period (∼10 weeks), a massive increase of pyramidal tract fibers, completion of pyramidal decussation, accumulation of pyramidal tract at the cervical levels of the spinal cord by 17 GW, reaching the lower thoracic chord by 19 GW and the lumbosacral cord by 29 GW [28] (Fig. 3a). Thus, the time window between 28.5 and 30.5 GW, i.e., the period during which we observed significant differences in AMT between upper and lower extremities, corresponds to the time window during which corticospinal and bulbospinal pathways for upper extremities are establishing connectivity or myelinating while corticospinal pathways for lower extremities are only reaching corresponding lumbar levels. Future studies that more densely sample the time period of this transition and techniques to separate spontaneous movements from cortically driven movements are planned to further explore this hypothesis.

The number of pyramidal tract fibers that decussate shows inter-individual differences [29] as well as asymmetry [compared to the left side, the right side of the cervical region of the spinal cord receives more pyramidal tract fibers from both cerebral hemispheres [30]]. It is generally accepted that hand preference develops during infancy. However, several reports focusing on the behavior of the fetus in utero suggest the development of the handedness around 10 GW. Hepper et al. [31, 32, 33] reported that by 13 GW, 90% of 274 fetuses studied preferred sucking their right thumb and that this hand preference persisted later in life. Despite these known asymmetries in the volume of the pyramidal tract and observations of fetal behavior, our results did not show left-right differences in AMT. Perhaps, larger studies and more sophisticated characterization of the kinematic movement (e.g., angular velocity) will be able to capture potential asymmetries in the character of the limb movement.

We also observed a decline in the AMT after peaking at 30 GW, a trend that was similar for both upper and lower extremities (Fig. 3b). This decline coincides with the increased inhibitory influence of the corticospinal tract [34] but also an increase in restriction in fetal movement caused by a decline in the relative volume of amniotic fluid [35]. Similar qualitative decreases have been described in the literature, but we provide the first quantitative assessment. Thus in future studies assessing potential abnormalities of fetal motion, such as in Chiari II malformations, expected age-related decreases can be regressed out.

Finally, we also identified a period between 35.5 and 36.5 GW characterized by a significantly longer (39.4%) AMT of lower extremities. This period coincides with a well-known transition in character of general movements that change into the more slow and forceful “writhing” general movements [3]. As noted above, additional methods to quantitatively characterize these movements are planned.

There are several limitations of our study. First, since the temporal BOLD MRI resolution is approximately 3 s, the shorter duration motion such as rapid jerks cannot be captured. Second, due to the low spatial resolution and the fact that only joints were labeled (key points), additional details of motion, such as the motion of the fetal hands/feet and fingers/toes, were not captured. Currently, only the presence or absence of movement is captured. Ongoing research aims to increase the ability to track hands and feet as well as to characterize more complex fetal movements. Finally, the generalizability of the results is limited given that MRIs were acquired at a single institution using one MRI scanner. Further prospective harmonized multi-site studies are needed to corroborate the findings presented here and are underway. Taken all together, our results suggest that ML analysis of fetal MRI could be used for non-biased assessment of fetal movement and potentially help in the development of biomarkers of outcome in conditions affecting maternal-fetal oxygen transfer (e.g., placental pathology) or neuromotor development (e.g., Chiari II malformations and many neurological disorders such as in utero injury and malformations).

## Conclusion

In conclusion, our results suggest that ML analysis of fetal MRI can be used to quantify in utero movement of the fetal extremities. We showed that the amount of fetal motion is likely determined by fetal anatomy (anatomical and physiological maturation of the nervous system) and is influenced by maternal and placental physiology (oxygen delivery). Future research using fetal movement as a biomarker of fetal development should take into consideration fetal, maternal, and placental anatomy and physiology.

## Statement of Ethics

Written informed consent was obtained from all subjects. This study protocol was reviewed and approved by Boston Children's Hospital IRB, approval number 00012586. All methods were carried out in accordance with institutional guidelines and regulations.

## Conflict of Interest Statement

The authors have no conflicts of interest to declare.

## Funding Sources

This work was supported by Ralph Schlaeger Foundation (L.V.) and National Institute of Health P41EB015902 (P.G.), NIH U01EB028236 (P.E.G., E.A., P.G.), NIH R01HD100009 (P.E.G., E.A., P.G.), and NIH U01HD087211 (P.E.G., E.A., P.G.).

## Author Contributions

The authors' contributions are as follows: design of the study (L.V., E.A.-T., E.A., P.G., and P.E.G), data management and collection (C.Z. and E.H.), image analysis (E.A.-T. and J.X.), MRI image interpretation (J.E., C.B., S.C., and P.E.G), statistical analysis (H.F.), and interpretation of the results (L.V., E.A.-T., W.B., and P.E.G.). All authors contributed to the preparation of the manuscript.

## Data Availability Statement

Anonymized processed data are available from the corresponding author upon reasonable request.

## Supplementary Material

Supplementary data

Video 1 Supplemental Video

## Figures and Tables

**Table 1:** Maternal, placental, and fetal characteristics

Maternal factors	
Maternal age [mean ± SD], years	32.98±3.45
Missing [*N*/TOTAL (%)]	1/52 (1.9)
BMI [mean ± SD]	29.99±7.19
Missing [*N*/TOTAL (%)]	9/52 (15.4)
Parity [*N*/TOTAL (%)]	
Multipara	[20/52 (38.46)]
Primipara	[24/52 (46.15)]
Missing	[9/52 (17.31)]
Position of the mother during MRI scan [*N*/TOTAL (%)]	
Multiple positions	[24/52 (46.15)]
Left lateral	24/24 (100)
Supine	19/24 (79.17)
Right lateral	1/24 (4.17)
Tilt	1/24 (4.17)
Single position	[28/52 (53.87)]
Left lateral	26/28 (92.86)
Supine	1/28 (3.57)
Right lateral	1/28 (3.57)
Tilt	0/28 (0)
Placental factors	
Location of the placenta [*N*/TOTAL (%)]
Anterior	[26/52 (50)]
Posterior	[24/52 (46.15)]
Previa	[1/52 (1.92)]
Superior	[1/52 (1.92)]
Fetal factors	
Fetal age in GW [mean ± SD]	32.08±2.71
Sex [*N*/TOTAL (%)]	
Male	[31/52 (59.62)]
Female	[21/52 (40.38)]
Fetal presentation [*N*/TOTAL (%)]	
Vertex	[42/52 (80.77%)]
Breech	[8/52 (15.28%)]
Transverse	[2/52 (3.85%)]
Fetal position [*N*/TOTAL (%)]	
Left occiput	[25/52 (48.08)]
Right occiput	[15/52 (28.85)]
Left sacrum	[4/52 (7.69)]
Right sacrum	[5/52 (9.62)]
Occiput anterior	[3/52 (5.77)]

**Table 2:** Parameter estimates of the fitted mixed-model ANOVA for the AMT

Effect	*p* value	Contrast	Estimate percent [%]	Standard error	95% confidence intervals		*p* value	FDR
Maternal factors								
MRI position of the mother	0.29	Left lateral-supine	65.3	48.7	−3.4	182.9	0.07	0.392
		Supine-tilt	−52.7	84.1	−94.4	296.0	0.47	1.000
		Right lateral-supine	58.9	178.8	−66.9	661.9	0.55	1.000
		Right lateral-tilt	−21.9	135.2	−90.3	530.8	0.81	1.000
		Left lateral-tilt	−24.9	181.8	−94.2	869.6	0.82	0.982
		Left lateral-right lateral	4.0	110.6	−76.9	369.2	0.96	0.957
Parity	0.90	First pregnancy-multipara	14.8	39.6	−35.9	105.4	0.64	1.000
		First pregnancy-unknown	10.6	77.7	−61.0	214.1	0.85	1.000
		Unknown-multipara	3.8	71.7	−63.0	190.6	0.94	0.944
Oxygen levels	**<0.0001**	Hyperoxia-normoxia	20.7	3.6	13.9	27.9	**<0.0001**	**0.000**

Placental factors								
Placental position	0.16	Anterior-superior	−96.2	13.6	−99.8	−25.2	**0.03**	0.189
		Posterior-superior	−95.3	16.9	−99.8	−6.3	0.05	0.136
		Previa-superior	−91.4	51.8	−99.8	293.0	0.21	0.416
		Anterior-previa	−55.4	105.3	−95.9	380.7	0.51	0.758
		Anterior-posterior	−18.8	30.7	−56.7	52.2	0.52	0.619
		Posterior-previa	−45.1	122.7	−94.5	449.1	0.61	0.609

Fetal factors								
Fetal position	0.19	Left occiput-right occiput	−42.1	21.5	−68.8	7.5	0.08	0.416
		Occiput anterior-right occiput	−76.7	28.0	−95.0	9.4	0.06	0.648
		Occiput anterior-right sacrum	−48.4	81.2	−91.9	229.5	0.48	0.806
		Left sacrum-occiput anterior	193.2	455.1	−53.3	1,741.8	0.25	0.502
		Left occiput-occiput anterior	148.8	284.6	−44.2	1,010.5	0.23	0.580
		Right occiput-right sacrum	121.5	180.2	−31.1	612.0	0.18	0.606
		Left sacrum-right occiput	−31.8	55.3	−78.7	118.5	0.52	0.742
		Left occiput-left sacrum	−15.1	67.1	−72.9	166.1	0.78	0.778
		Left occiput-right sacrum	28.3	113.5	−63.0	344.6	0.69	0.772
		Left sacrum-right sacrum	51.1	171.3	−65.8	568.1	0.59	0.732
Joint distance	**<0.0001**	Distal-proximal	49.3	5.5	39.1	60.4	**<0.0001**	**0.000**
		Middle-proximal	23.8	4.6	15.3	32.9	**<0.0001**	**0.000**
		Distal-middle	20.6	4.5	12.4	29.5	**<0.0001**	**0.000**
Interaction								
[GA*extremity]	**<0.0001**	27 * [lower-upper]	94.4	56.1	18.3	219.6	**0.009**	0.053
		28 * [lower-upper]	−10.6	8.1	−24.6	6.0	0.20	0.392
		29 * [lower-upper]	−39.3	6.8	−50.7	−25.3	**<0.0001**	0.000*
		30 * [lower-upper]	−25.7	12.2	−44.9	0.2	**0.05**	0.154
		31 * [lower-upper]	−10.7	9.0	−26.1	7.9	0.24	0.363
		32 * [lower-upper]	6.9	8.3	−7.6	23.8	0.37	0.443
		33 * [lower-upper]	15.8	10.4	−2.2	37.2	0.09	0.211
		34 * [lower-upper]	6.7	7.3	−6.3	21.5	0.33	0.439
		35 * [lower-upper]	−5.0	10.5	−22.7	16.8	0.63	0.626
		36 * [lower-upper]	39.4	19.4	7.9	80.0	**0.011**	**0.044**
		37 * [lower-upper]	12.3	18.2	−16.4	50.9	0.44	0.481
		38 * [lower-upper]	−27.0	21.2	−55.7	20.3	0.22	0.371
[GA*side]	**0.05**	30 * [left-right]	−34.4	10.8	−51.3	−11.5	**0.006**	0.069
Sex	0.97	Female-male	1.0	35.5	−44.1	82.5	0.97	0.974

Gestational age in weeks (GA). Bolded and italicized: significant. Bolded: significant after FDR (*).
