# Supplementary material for: Cross-Sectional Observational Study of Typical in utero Fetal Movements Using Machine Learning
Source: Dev Neurosci. 2022 Dec 20;45(3):105–14. doi: 10.1159/000528757 (PMC10233700; doi:10.1159/000528757)
Supplement: Supplementary file 1 — Supplementary data [file dne-0045-0105-s01.docx]

# Supplementary Information

## Movie

### Movie 1. Real-time measurements illustrate the difference in Absolute Movement Time between lower and upper extremities before 30 GW in one fetus.

A: an example slice of the MR volume. B: the fetal pose generated from labeled key points. C: 3D masks of the fetal body and uterus. D: the change of velocity over time, shaded regions indicate velocities are greater than the threshold.
